# Dietary iron modulates gut microbiota and induces SLPI secretion to promote colorectal tumorigenesis

**DOI:** 10.1080/19490976.2023.2221978

**Published:** 2023-06-13

**Authors:** Chen Liu, Junli Gong, Qiang Zhang, Guangyuan Chen, Shengmei Yin, Zhanhao Luo, Wanyi Zeng, Jing Yu, Ping Lan, Zhen He

**Affiliations:** a Department of Colorectal Surgery, The Sixth Affiliated Hospital of Sun Yat-Sen University, Guangzhou, Guangdong, China; b Department of General Surgery, The Sixth Affiliated Hospital of Sun Yat-Sen University, Guangzhou, Guangdong, China; c Guangdong Provincial Key Laboratory of Colorectal and Pelvic Floor Diseases, Guangdong Institute of Gastroenterology, the Sixth Affiliated Hospital, Sun Yat-Sen University, Guangzhou, Guangdong, China; d Key Laboratory of Human Microbiome and Elderly Chronic Diseases, Ministry of Education, Guangzhou, Guangdong, China; e School of Medicine, Sun Yat-Sen University, Guangzhou, Guangdong, China

**Keywords:** Excessive dietary iron, gut microbiome, colorectal cancer, secretory leukocyte protease inhibitor, tumorigenesis, epithelium

## Abstract

Dietary iron intake is closely related to the incidence of colorectal cancer. However, the interactions among dietary iron, gut microbiota, and epithelial cells in promoting tumorigenesis have rarely been discussed. Here, we report that gut microbiota plays a crucial role in promoting colorectal tumorigenesis in multiple mice models under excessive dietary iron intake. Gut microbiota modulated by excessive dietary iron are pathogenic, irritating the permeability of the gut barrier and causing leakage of lumen bacteria. Mechanistically, epithelial cells released more secretory leukocyte protease inhibitor (SLPI) to combat the leaked bacteria and limit inflammation. The upregulated SLPI acted as a pro-tumorigenic factor and promoted colorectal tumorigenesis by activating the MAPK signaling pathway. Moreover, excessive dietary iron significantly depleted *Akkermansiaceae* in the gut microbiota; while supplementation with *Akkermansia muciniphila* could successfully attenuate the tumorigenic effect from excessive dietary iron. Overall, excessive dietary iron perturbs diet – microbiome–epithelium interactions, which contributes to intestinal tumor initiation.

## Introduction

Colorectal cancer (CRC) is one of the most prevalent malignant tumors worldwide, with the third-highest incidence and mortality.^1^ With continued industrialization, global dietary patterns have gradually become more western, a diet that is characterized by red meat, fatty acids, and abundant carbohydrates.^2–4^ Generally, sporadic CRC is largely attributed to diets that are high in fat and carbohydrate,^5^ which can lead to oxidative stress^6,7^ and microbiota dysbiosis.^8,9^ Besides, epidemiological studies have demonstrated a close relationship between dietary iron intake and CRC incidence in several ethnic groups.^10–16^ However, the mechanism by which excessive dietary iron promotes carcinogenesis remains poorly understood.

Interactions between the host, gut microbiota, and nutrition are complex.^17,18^ Recent research has identified the effect of dietary iron on gut microbiota.^19,20^ Although iron is necessary for the multiplication and localization of bacteria,^21^ the acquisition of iron varies among bacterial strains.^22^ Meanwhile, the feces of individuals with CRC, which are characterized by an increase in the tumorigenesis bacterial strain and leakage of probiotics, caused an increased incidence of CRC in mice.^23,24^ However, the cause of these changes in the microbiota of patients with CRC is unclear. Nevertheless, these results suggest that dietary iron can modulate the composition of gut microbiota and thus accelerate tumorigenesis.

Secretory leukocyte protease inhibitor (SLPI) is a cytokine predominantly generated by epithelial cells that inhibits leukocyte protease activation and functions as an antimicrobial peptide.^25,26^ SLPI is a highly conserved pleiotropic protein that regulates the inflammatory response, inhibits tissue destruction, and protects the host from infection.^27^ These characteristics enable tumor cells to utilize SLPI to establish an immunosurveillance microenvironment.^28^ Recent studies have reported the upregulation of SLPI in various malignant tumors.^29–31^ SLPI can also accelerate tumor development,^32^ promote tumor metastasis,^33^ and induce vasculogenic mimicry.^34^ However, it remains unclear whether gut microbiota affect carcinogenesis by regulating SLPI expression.

In this study, we aim to elucidate the role of gut microbiota in CRC related to excessive dietary iron. Using a mouse CRC model, we identify the effect of iron on microbiota and prove that iron-modulated microbiota irritates epithelial permeability and accelerates CRC carcinogenesis. This results in the induction of SLPI in epithelial cells, which triggers the oncogenic MAPK signaling pathway, thereby increasing colonocyte proliferation.

## Results

### Excessive iron intake promotes CRC

**Figure 1.**
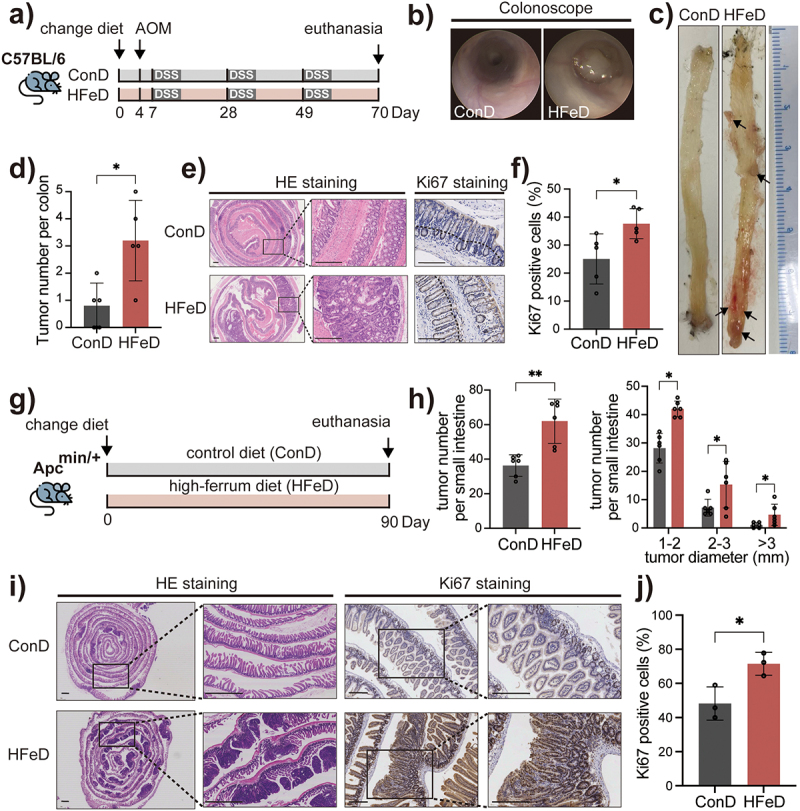
Excessive dietary iron increases colorectal tumorigenicity in mice. (a) Schematic overview of the AOM/DSS-induced colorectal cancer model. Mice were fed with control diet (ConD) or high-iron diet (HFeD) for 10 weeks. AOM (10 mg/kg) was injected intraperitoneally at day 4. Mice were sacrificed at the end of week 10 (ConD group, *n* = 6; HFeD group, *n* = 5). (b – c) Representative images of colonoscope and colon when sacrificed. (d) Tumor numbers in ConD and HFeD mice. (e) Representative images of H&E staining and Ki67-positive cells of colon sections in the ConD group and HFeD group. Scale bars, 250 μm. (f) Quantitation of Ki67 expression in colons. (g) Schematic overview of the transgenic Apc^min/+^ cancer model. Mice were fed ConD or HFeD diets for 13 weeks. (h) Tumor number (left) and tumor size (right) in ConD and HFeD mice. (i) Representative images of H&E staining (Scale bars, 1,000 μm) and Ki67-positive cells (Scale bars, 200 μm) of small intestine sections in the ConD group and HFeD group. (j) Quantitation of Ki67 expression in the small intestines. Data are expressed as the mean ± SD. Each data point represents one mouse. Statistical significance was determined by unpaired Student’s t-test and ordinary one-way ANOVA with Tukey’s multiple comparisons. *p < .05, **p < .01. AOM, azoxymethane. DSS, dextran sulfate sodium salt.

To demonstrate the effect of dietary iron on the pathogenesis of CRC, we first established an azoxymethane/dextran sulfate (AOM/DSS)-induced mouse CRC model^35^ using male C57BL/6 mice fed either a control diet of basic AIN93G (ConD) or a high-iron diet of AIN93G with 1,000 mg/kg of iron (HFeD), which is similar to the iron intake of a western diet (Figure 1a). In addition, we established the same model with mice fed either pure AIN93G (ConD) or a low-iron diet of AIN93G with 250 mg/kg of iron (LFeD), which represents the recommended iron intake (Figure S1A). Interestingly, HFeD mice had more macroscopic tumors (Figure 1b,e), whereas LFeD and ConD groups had similar tumor numbers (Figure S1B – D). Colon sections stained with Ki67 revealed a greater number of positive epithelial cells in the HFeD group (Figure 1e,f), indicating that excessive dietary iron may result in increased cell proliferation.

To further investigate the effect of excessive dietary iron on tumorigenesis, we established a transgenic Apc^min/+^ mouse model with ConD and HFeD (Figure 1g). Consistent with the results of the AOM/DSS model, we observed higher tumor numbers and a larger tumor size in HFeD mice (Figure 1h). Along with hematoxylin and eosin (H&E) staining, Ki67 staining demonstrated that HFeD mice possessed a greater number of Ki67-positive epithelial cells, indicating an improved proliferative state (Figure 1i,j). These data indicate that excessive dietary iron intake promotes carcinogenesis.

### Gut microbiota is indispensable for excessive dietary iron to promote CRC

**Figure 2.**
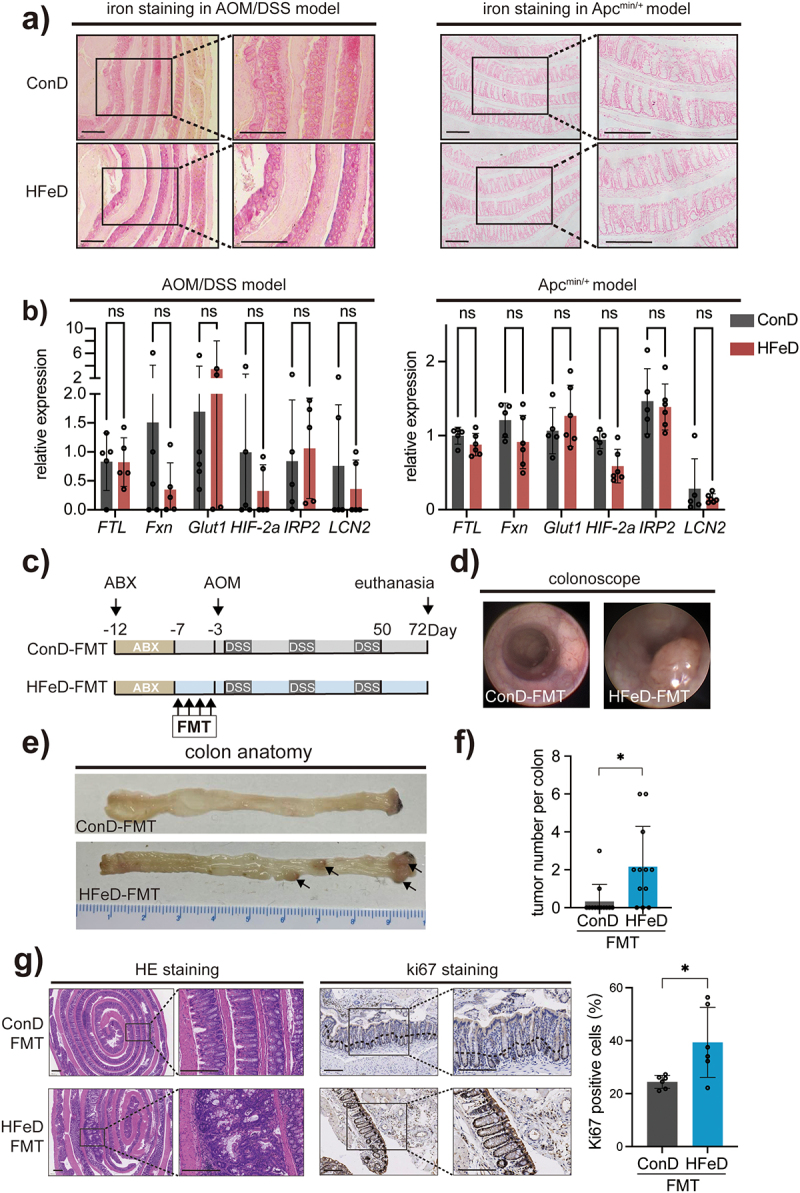
Microbiota modulated by excessive dietary iron increases colorectal tumorigenicity in mice. (a) Representative images of enhanced Perls’ staining (Scale bars, 400 μm) of small intestine sections in the ConD group and HFeD group (left, AOM/DSS model; right, Apc^min/+^ model). (b) Gene expression of FTL, Fxn, Glut1, HIF −2a, IRP2, and LCN2 in ConD and HFeD mice (left, AOM/DSS model; right, Apc^min/+^ model). (c) Schematic overview of the AOM/DSS-induced cancer model. Mice were treated with an antibiotics cocktail for five days and later underwent washed fecal microbiota transformation for four consecutive days. AOM (10 mg/kg) was injected intraperitoneally at day 0. Mice were sacrificed at the end of week 10 (ConD group, *n* = 12; HFeD group, *n* = 12). (d – e) Representative images of colonoscope and colon when sacrificed. (f) Tumor number in ConD and HFeD mice. (g) Representative images of H&E staining and Ki67-positive cells of colon sections in the ConD-FMT group and HFeD-FMT group (Scale bars, 400 μm). Quantitation of Ki67 expression in the colons (right). Data are expressed as the mean ± SD. Each data point represents one mouse. Statistical significance was determined by unpaired Student’s t-test and multiple unpaired Student’s t-test. *p < .05.

Dietary iron was predominantly absorbed by epithelial cells.^36^ Recently, studies have shown that an overload of intracellular iron can irritate the oxidative balance, causing tumorigenesis.^37,38^ To confirm whether excessive dietary iron lead to the enrichment of intracellular iron, Prussian blue iron staining were utilized to examine intracellular iron of small intestine. To our surprise, no significant changes in intracellular iron were observed in either HFeD or ConD mice (Figure 2a). In addition, quantitative real-time PCR (qPCR) was used to map the epithelial iron homeostasis landscape. Genes involved in iron storage, transit, and utilization, such as *ftl, fxn, glut1, hif−2a, irp2, and lcn2*, were found to be invariant between groups, demonstrating that iron hemostasis inside epithelial cells was still stable even under excessive dietary iron (Figure 2b). These findings indicated that the carcinogenesis role of excessive dietary iron is independent of intracellular iron accumulation.

Research on microbiome-host interactions has demonstrated that gut microbiota are dynamic^39^ and has a significant effect on the biological activities of epithelial cells.^40^ To determine whether bacteria contributes to the tumorigenic capacity of excessive dietary iron, we treated C57BL/6 mice with periodic antibiotic cocktails in combination with either ConD or HFeD (Figure S2a). The results showed that excessive dietary iron was less effective in promoting colorectal carcinogenesis, when gut microbiota were depleted by antibiotics (Figure S2e); this finding was also histologically verified (Figure S2c). Simultaneously, colon sections stained with Ki67 revealed a similar number of positive cells in ConD mice (Figure S2c-d). The combination of microbiota and dietary iron had a massive impact on colorectal carcinogenesis; however, dietary iron alone appeared to have no effect, indicating that dietary iron-modified microbiota might be hazardous. Additionally, to investigate the contribution of the microbiome to tumorigenesis, we performed fecal microbiota transplantation (FMT) in SPF male C57BL/6 mice treated with AOM/DSS (Figure 2c). We conducted colonoscopy before euthanasia and observed an increase in macroscopic tumors in mice fed with HFeD-modulated microbiota (Figure 2d). As expected, more clonal tumors were observed in mice that received HFeD-modified microbiota (Figure 2e,f). Colon sections were histologically examined. In addition to the increased tumor number, a higher number of Ki67-positive cells were identified in the colon sections of HFeD-FMT mice than in those of ConD-FMT mice (Figure 2g). Taken together, our results suggest that excessive dietary iron promotes colorectal tumorigenesis by altering gut microbes.

### Gut microbiota modulated by HFeD destroys the gut barrier

**Figure 3.**
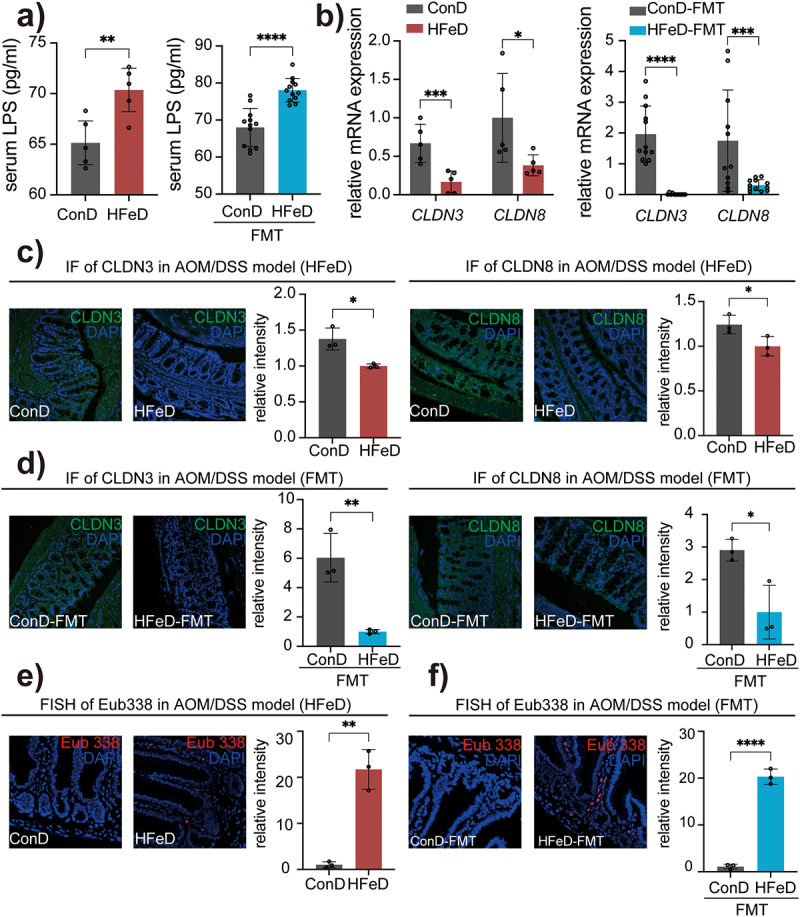
Excessive dietary iron and modulated microbiota impair gut barrier function. (a) LPS concentration in the serum of ConD and HFeD mice (left) or ConD-FMT and HFeD-FMT mice (right). (b) Gene expression of CLDN3 and CLDN8 in ConD and HFeD mice (left) or ConD-FMT and HFeD-FMT mice (right). (c) Representative images of immunofluorescence staining and quantitation of CLDN3 and CLDN8 in AOM/DSS model. (d) Representative images of immunofluorescence staining and quantitation of CLDN3 and CLDN8 in AOM/DSS model with FMT. (e) Staining of total bacteria in the AOM-treated model by fluorescence in situ hybridization in ConD and HFeD mice. (f) Staining of total bacteria in the AOM-treated model by fluorescence in situ hybridization in ConD-FMT and HFeD-FMT mice. Each data point represents one mouse. Data are expressed as the mean ± SD. Statistical significance was determined by unpaired Student’s t-test. *p < .05, **p < .01, ***p < .001, ****p < .0001.

Gut microbiota dysbiosis has a critical impact on increasing gut permeability and epithelial barrier, and gut barrier dysfunction has been regarded to be closely related to the occurrence of CRC.^41,42^To investigate the impact of HFeD and its modulated microbiota on gut barrier function, we measured serum lipopolysaccharide (LPS) concentrations in mice. As expected, the distribution level of LPS was significantly increased in mice fed with HFeD as well as mice receiving HFeD-modulated microbiota (Figure 3a). We then performed transcriptome sequencing of mice colon tissue. Consistently, cell adhesion molecules were significantly altered in the KEGG pathway analysis (Figure S3a), and these results were furtherly verified by quantitative real-time PCR (qRT-PCR) detection (Figure S3b). We identified that the expression of Cldn3 and Cldn8 (key proteins required to sustain tight conjunctions and markers of gut barrier integrity)^43,44^ was significantly reduced in HFeD mice and HFeD-FMT mice through qRT-PCR (Figure 3b) and IF (Figure 3c,d), indicating that bacteria regulated by HFeD are detrimental to the gut barrier structure. To further analyze the repercussions of enhanced epithelial permeability, we detected the bacteria within the epithelial layer of mouse intestinal using a broad-spectrum bacterium probe (EUB338) via fluorescent in situ hybridization (FISH). Concurrent with gut barrier dysfunction, the migration of bacteria to the deep mucosa was considerably elevated in HFeD mice and HFeD-FMT mice (Figure 3e,f). In summary, these findings revealed that HFeD-modified bacteria could lead to the gut epithelial barrier disruption, which might play a driving role in promoting the occurrence of CRC.

### Modulated microbiota activate the MAPK signaling pathway by inducing SLPI

**Figure 4.**
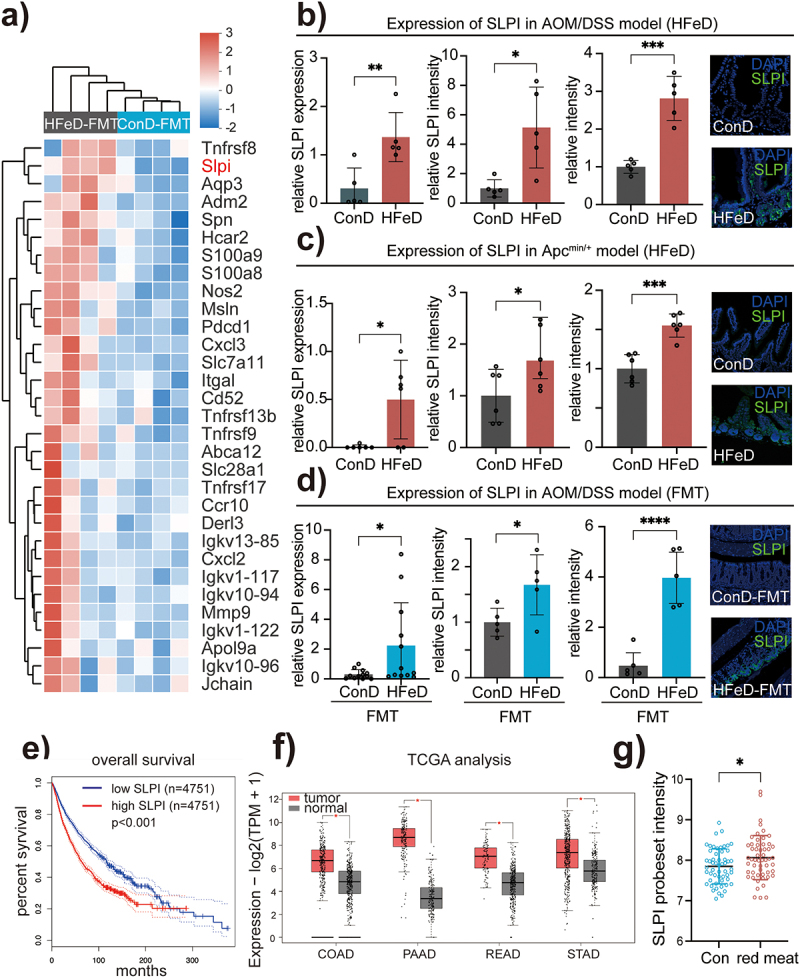
Excessive dietary iron and modulated microbiota induce SLPI expression. (a) Heatmap derived by Reactome annotation showing genes involved in immune regulation pathway. (b) Gene expression (left), protein expression (middle) and quantitation of immunofluorescence staining (right) of SLPI in ConD and HFeD mice (AOM/DSS model). For protein expression analysis and immunofluorescence staining, we randomly selected five mice from each group for analysis. (c) Gene expression (left), protein expression (middle) and quantitation of immunofluorescence staining (right) of SLPI in ConD and HFeD mice (Apc^min/+^ model). For protein expression analysis and immunofluorescence staining, we randomly selected six mice from each group for analysis. (d) Gene expression (left), protein expression (middle) and quantitation of immunofluorescence staining (right) of SLPI in ConD-FMT and HFeD-FMT mice (AOM/DSS model). For protein expression analysis and immunofluorescence staining, we randomly selected five mice from each group for analysis. (e) Kaplan–Meier plot showing survival of patients with tumors (*N* = 9,502) stratified for low and high SLPI expression in primary tumors. (f) Transcription levels of SLPI in colon adenocarcinoma (COAD), pancreatic adenocarcinoma (POAD), rectum adenocarcinoma (READ), stomach adenocarcinoma (STAD), and normal tissue (GEPIA). (H) Gene expression of SLPI in humans that consume more red meat (*n* = 54) and less red meat (*n* = 54). Each data point represents one mouse (b-d). Data are expressed as the mean ± SD. Statistical significance was determined by unpaired Student’s t-test. *p < .05, **p < .01, ***p < .001, ****p < .0001.

To elucidate the specific mechanism by which HFeD-modulated microbiota accelerates clonal carcinogenesis, we further analyzed the transcriptome sequencing of mice receiving either HFeD or ConD-modulated microbiota. A clustered heatmap based on Reactome annotation showed that SLPI expression was dramatically increased in HFeD-FMT mice (Figure 4a). Moreover, qRT-PCR, immunoblotting, and immunofluorescence assays confirmed the enhanced SLPI expression in mice administered HFeD from both AOM/DSS model and Apc^min/+^ model (Figure 4b,c; S4a-c; S4g). Similarly, mice that underwent FMT with either HFeD also showed increased expression of SLPI at both the transcription and protein levels (Figure 4d; S4a; S4d; S4g) but not in antibiotics treated mice (Figure S4e-f). Next, we evaluated the TCGA database to determine the relationship between overall survival and SLPI expression in pan-cancer (Figure 4e), which concluded that patients with high SLPI expression had poor survival prognosis. Increased expression of SLPI was also found in multiple human cancer types (Figure 4f). Similarly, we investigated whether human consumption of red meat was associated with increased SLPI expression. Surprisingly, SLPI expression was considerably increased in subjects who consumed more red meat (Figure 4g),^45^ which corroborated our findings from the animal experiments. Taken together, we demonstrated that HFeD-modulated microbiota induced SLPI expression.

**Figure 5.**
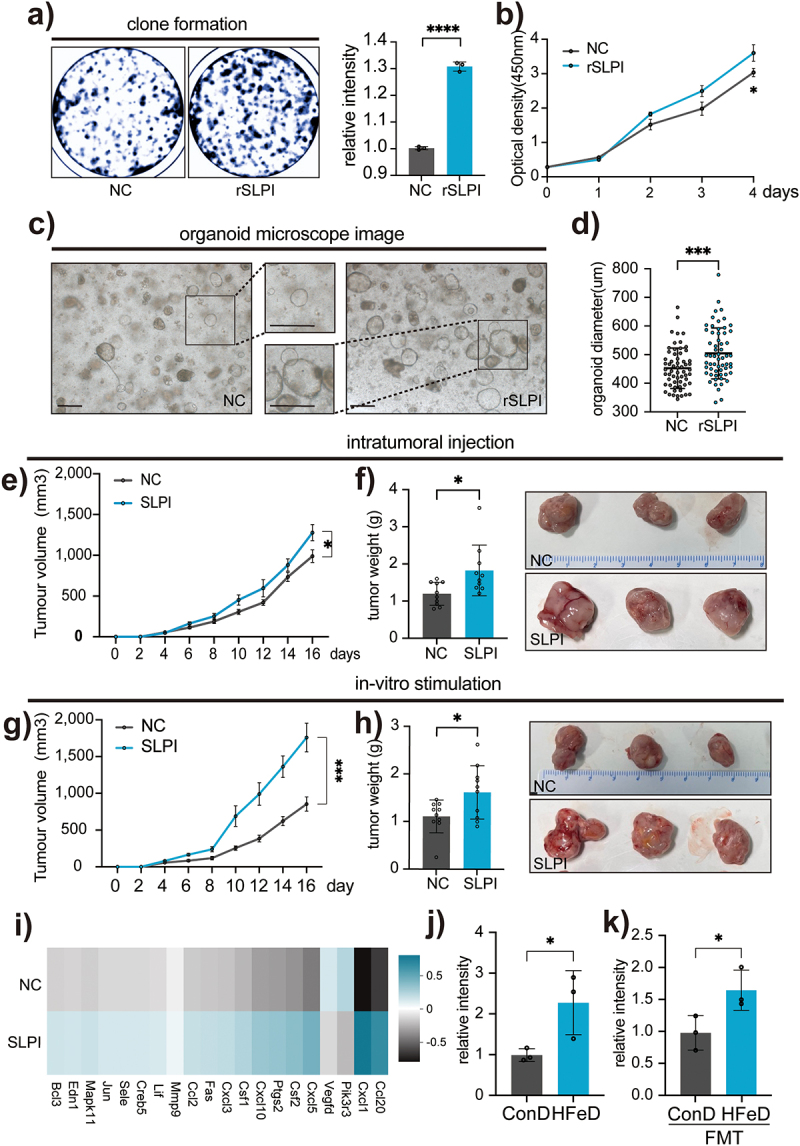
SLPI promotes tumor development both in vivo and in vitro. (a) Representative images and statistical chart of clone formation of CT26 stimulated with PBS (NC) or SLPI (rSLPI). (b) CCK8 assay of CT26 stimulated with PBS (NC) or SLPI (rSLPI). (c – d) Representative images (c) and statistical chart (d) of human-derived tumoroid stimulated with PBS (NC) or SLPI (rSLPI) for seven days. Scale bars, 400 μm. (e – f) in vivo primary tumor growth (e), representative images and statistical chart (f) in mice subcutaneously injected with CT26 and intratumorally injected with PBS (NC) or SLPI. Each data point represents one mouse. (g – h) in vivo primary tumor growth (g), representative images, and statistical chart (h) in mice subcutaneously injected with CT26 pre-treated with PBS (NC) or SLPI for two days. Each data point represents one mouse. (i) Heatmap of genes in the MAPK signaling pathway. (j) Protein expression of phosp-SAPK/JNK in ConD and HFeD mice or ConD-FMT and HFeD-FMT mice determined by western blot. Data are expressed as the mean ± SD. Statistical significance was determined by unpaired Student’s t-test. *p < .05, **p < .01, ***p < .001, ****p < .0001.

Next, we intend to explore whether SLPI play a role in promoting tumor growth. Colony formation and CCK−8 experiments showed that CRC cells treated with recombinant SLPI had a higher proliferation capacity (Figure 5a,b). Then, we cultivated CRC patient-derived tumoroids as described previously,^46^ then stimulated them with recombined human SLPI for three consecutive days. The results suggested that SLPI hastened tumoroid development (Figure 5c,d). For the *in vivo* experiments, subcutaneous tumors intratumorally treated with mouse SLPI exhibited higher growth rates and larger tumors (Figure S4h; 5e-f). We then stimulated CRC cells with SLPI for three days before subcutaneously injecting them into BalB/c mice (Figure S4i). An increased tumor growth rate was observed in those stimulated by SLPI (Figure 5g), which resulted in greater tumor weight (Figure 5h). We also generated *slpi*-depleted CRC cells using shRNA, then performed CCK−8 and subcutaneous tumor formation assays. As expected, the depletion of slpi significantly reduced tumor growth both in vitro and in vivo (Figure S4j-m). In summary, we demonstrated that SLPI played a crucial role in promoting tumor growth.

To determine the mechanism by which SLPI promotes tumorigenesis, we stimulated CT26 cells with SLPI and performed transcriptome sequencing. PCA was also used to determine whether SLPI stimulation resulted in distinct transcriptional changes (Figure S4n). We discovered that the differences primarily occurred in the MAPK transduction pathway, a well-known pathway involved in cancer (Figure S5a; 5i). We then examined the expression of three traditional MAPK signaling pathways and found that only phosphorylated SAPK/JNK was significantly activated in SLPI-treated CRC cells (Figure S5b-c). We found that the SAPK/JNK pathway was also upregulated in HFeD mice (Figure 5j; S5d) and mice administered HFeD-modulated microbiota (Figure 5k; S5e). These results suggest that HFeD or microbiota modulated by HFeD induced SLPI, thereby promoting tumorigenesis by activating the SAPK/JNK signaling pathway.

### Excessive dietary iron significantly depletes Akkermansiaceae

**Figure 6.**
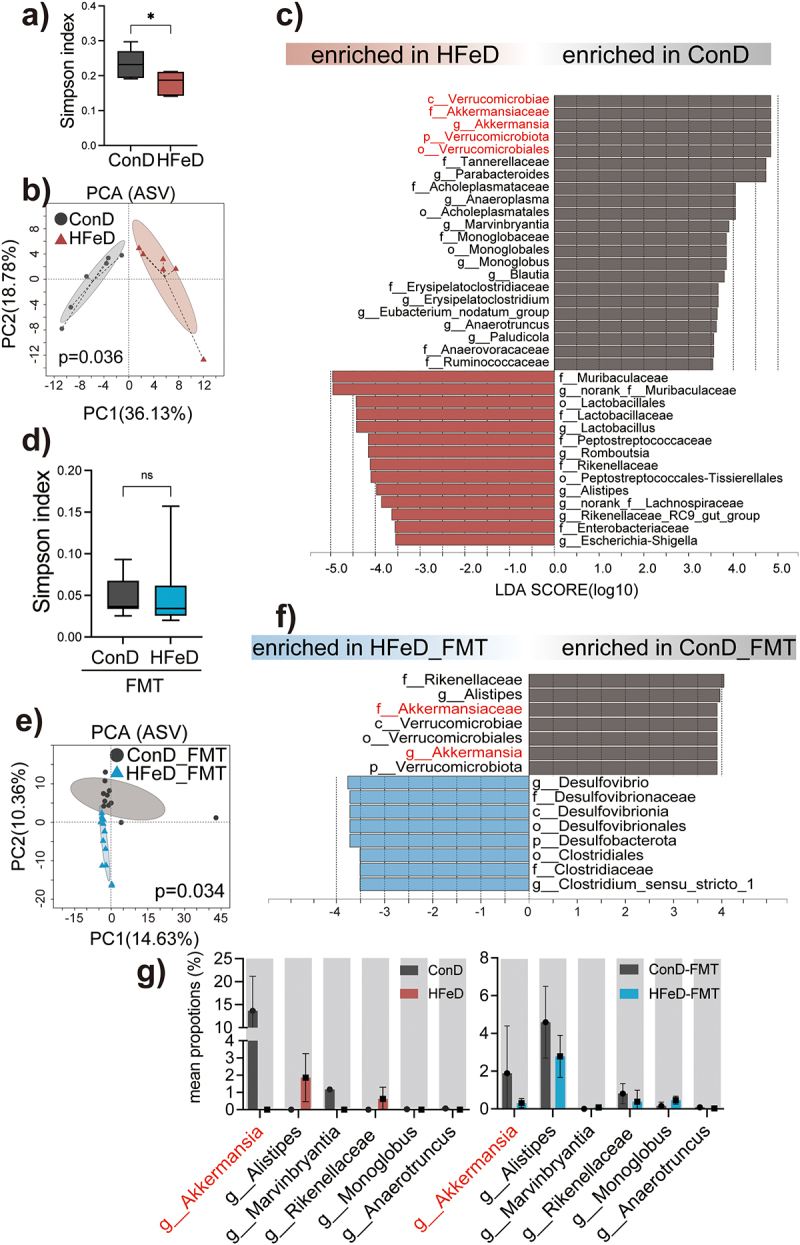
Excessive dietary iron significantly depletes *Akkermansiaceae*. (a) Alpha diversity (Simpson index) boxplot of mice in ConD (*n* = 6) and HFeD (*n* = 5) groups. (b) PCA comparing mouse microbial compositions in fecal samples between ConD and HFeD groups. (c) LDA score computed from features with differential abundance between ConD and HFeD groups; the criterion for feature selection was log LDA score > 3.5. (d) Alpha diversity (Simpson index) boxplot of mice in ConD-FMT (*n* = 11) and HFeD-FMT (*n* = 12) groups. (e) PCA comparing mouse microbial compositions in fecal samples between ConD-FMT and HFeD-FMT groups. (f) LDA score computed from features with differential abundance between ConD-FMT and HFeD-FMT groups; the criterion for feature selection was log LDA score > 3.5. (g) Statistical chart of the proportion of differentiated genus in ConD and HFeD groups or ConD-FMT and HFeD-FMT groups. Data are expressed as the mean ± SD. Statistical significance was determined by permutation multivariate analysis of variance (b,e) and unpaired Student’s t-test. *p < .05.

To detect the specific alterations in gut microbiota induced by HFeD, we conducted 16s rDNA sequencing on stool samples taken from ConD, LFeD, and HFeD mice in the AOM/DSS model. As predicted, food adjustments had an impact on the a-diversity (Figure 6a; Figure S6a & S7a). The geographical distribution of ConD-modulated microbiota was distinct from that of HFeD-modulated microbiota (Figure 6b) but overlapped with that of LFeD-modulated microbiota (Figure S6C-D). According to Linear discriminant analysis Effect Size (LefSe), the abundance of several potentially beneficial bacterial taxa, such as *Akkermansiaceae*^47^ and *Tannerellaceae*,^48^ was significantly reduced in HFeD mice; while, several potentially pathogenic bacteria, such as *Peptostreptococcaceae*,^49^ were significantly enriched in HFeD mice (Figure 6c), in contrast to LFeD-modulated microbiota (Figure S6e). These findings suggest that excessive dietary iron is detrimental to *Akkermansiaceae*.

To further confirm the tumorigenic microbiome pattern modified by HFeD, we collected fecal samples from mice administered either washed ConD-modulated microbiota or washed HFeD-modulated microbiota and performed 16s DNA sequencing. No significance in the alpha diversity was observed between the gut microbial community of HFeD-FMT and ConD-FMT mice (Figure 6d; Figure S6B, S7B); however, PCA could be discriminated (Figure 6e). In addition, we employed LefSe to examine the compositional changes in several bacterial taxa and discovered that *Akkermansiaceae* as well as *Rikenellaceae* were dramatically decreased in HFeD-FMT mice (Figure 6f,g). Taken together, these findings indicate that HFeD modulated the gut microbial community, and highlight that *Akkermansiaceae* might be crucial during HFeD-induced clonal tumorigenesis.

### Supplementation of Akkermansiaceae muciphila protects epithelial tumorigenesis against dietary iron

**Figure 7.**
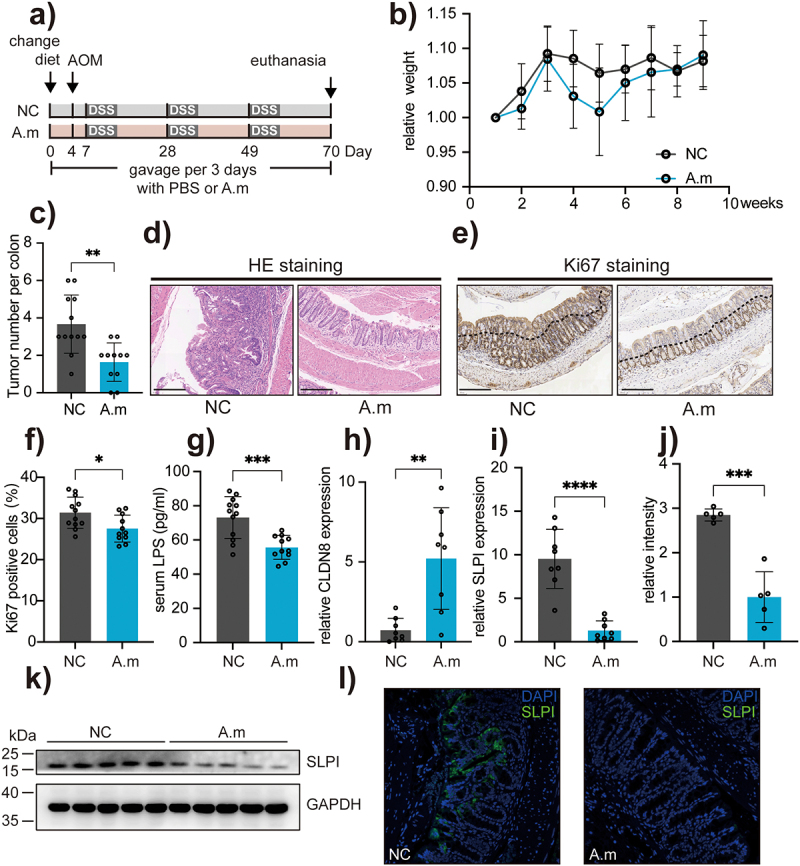
Supplementation of *Akkermansiaceae muciphila* protects epithelial tumorigenesis against dietary iron. (a) Schematic overview of the AOM/DSS-induced cancer model. Mice were gavaged every three days with PBS (NC) or A. muciphila for 10 weeks. AOM (10 mg/kg) was injected intraperitoneally at day 4. Mice were sacrificed at the end of week 10 (NC group, *n* = 12; A.M group, *n* = 11). (b) Weight changes are expressed as the mean change from the starting weight. (c) Tumor number in the mice of NC and A.M groups. (d – e) Representative images of H&E staining and Ki67-positive cells of colon sections in the NC and A.M groups. Scale bars, 200 μm. (f) Quantitation of Ki67 expression in the colons. (g) LPS concentration in the serum of mice from NC and A.M groups. (h) Gene expression of CLDN8 in mice from NC and A.M groups. (i) Gene expression of SLPI in mice from NC and A.M groups. (i – k) Protein expression of SLPI in mice from NC and A.M groups determined by western blot. (l) Protein expression of SLPI in NC and A.M mice determined by immunofluorescence staining. Data are expressed as the mean ± SD. Each data point represents one mouse. Statistical significance was determined by unpaired Student’s t-test. *p < .05, **p < .01, ***p < .001, ****p < .0001.

To investigate the potential protective effect of *Akkermansiaceae* against HFeD-induced tumorigenesis, we isolated *Akkermansiaceae* (specifically *Akkermansiaceae muciphila*) from mouse fecal samples and administered it to mice fed with the AOM/DSS model and HFeD via gavage every three days until euthanasia (Figure 7a). Our results indicated that *A. muciniphila* supplementation did not significantly affect mouse weight (Figure 7b), but it did significantly reduce the number of colon tumors (Figure 7c), which was confirmed by histological analysis using H&E staining (Figure 7d). Additionally, we measured epithelial proliferation using Ki67 staining and found that *A. muciniphila* supplementation reduced the staining area of Ki67 (Figure 7e,f). As mentioned above, HFeD could alter the microbiota and increase intestinal permeability. However, supplementation with *A. muciniphila* could partially restore gut barrier function, as evidenced by decreased serum LPS concentrations (Figure 7g) and increased expression of tight junction proteins (Figure 7h). We also measured SLPI expression, which we confirmed as promoting carcinogenesis in this study. Our results showed that *A. muciniphila* supplementation significantly reduced SLPI expression, as determined by qPCR and western blotting (Figure 7i-k). Finally, immunofluorescence analysis revealed a decrease in SLPI staining dots in mice that were gavaged with *A. muciniphila* (Figure 7l). Therefore, our findings indicated that supplementation with *A. muciniphila* protects against excessive dietary iron and upregulates SLPI expression by repairing gut barrier function.

## Discussion

In this study, we demonstrated that gut microbiota is indispensable to promote CRC under excessive iron intake. Gut microbiota modulated by HFeD could impair the gut barrier and up-regulate the expression of SLPI, which led to an activation of MAPK signaling in intestinal epithelial cells. Specifically, excessive dietary iron significantly depleted the distribution of *Akkermansiaceae*. Moreover, supplementary of *A. muciphila* attenuated the tumorigenesis effect from HFeD, suggesting the significant role of gut microbiota balance in protecting against HFeD-induced clonal tumorigenesis.

Several epidemiological studies have established correlations between dietary red meat and iron consumption and intestinal disease. For instance, Constante et al., (2017) reported that dietary heme, a component found in red meat, could lead to gut dysbiosis, worsen colitis, and potentiate the development of adenomas in mice^50^. Seiwert et al., (2021) revealed that a diet high in heme caused a reduction in α-diversity and persistent intestinal dysbiosis, which were associated with chronic gut inflammation and hyperproliferation of the intestinal epithelium^51^. In a separate study, Xiong et al., (2022) demonstrated that a high-iron diet played a role in regulating lipid metabolism and gut microbiota in mice^52^. However, the role of gut microbiota in excessive iron consumption-associated CRC remains unclear. In this study, we demonstrated that a high-iron diet promotes carcinogenesis in SPF mice, but not in mice treated by antibiotics cocktails. Additionally, our FMT experiment indicated that iron-induced modification of the gut microbiota plays a critical role in CRC, and that the gut microbiota is unneglectable in cases of excessive iron intake-associated CRC.

The intestinal epithelium functions as a barrier that limits interactions between luminal contents and the rest of the body, make it a fundamental function of human health^53^. The composition of gut microbiota is closely related to gut barrier function, and dysfunction of gut barrier could promote the development of CRC^48,49^. It has been suggested that dietary heme intake could increase gut inflammation and carcinogenesis by damaging the colon surface epithelium, characterized by slight damage to the duodenum with shortened and disarrayed villi, as well as the obviously sparse arrangement of duodenal microvilli. In this study, consistent with these results, we observed increased serum LPS and reduced expression of tight junction proteins in mice fed a high-iron diet. We also noticed an accumulation of intra-epithelial bacteria in the high-iron diet group, indicating that gut barrier dysfunction led to an invasion of lumen bacteria into the epithelium.

The leakage of bacteria into the epithelium can induce severe inflammation^54^. White blood cells, especially leucocytes, are chemotactic and activated to the infected area to secret proteases and pro-inflammatory agents to defend against the bacteria. SLPI is a protein secreted by the epithelium to inhibit the activity of leucocyte protease. Generally, gut epithelial cells secreted SLPI to maintain their own permeability and limit the density of epithelial inflammation^25,26^. The tumorigenic role of SLPI has been reported in breast cancer^54^, gastric cancer^55^ and CRC^29^. In this study, we observed that SLPI induced by HFeD-modulated bacteria was highly expressed in the intestine of HFeD-mice. This suggests that dysbiosis of gut microbiota can influence tumorigenesis by affecting the secretion of host immune regulatory proteins.

It has been reported that excess dietary iron could induce intestinal inflammation. However, unlike the decreased bacterial diversity in colitis mice model, a much higher diversity was observed in the HFeD-treated group, which has also been reported previously^56^. We speculate that the high-iron diet may have altered the symbiotic environment in the gut, leading to a significant imbalance in the original ecological niche. Excessive dietary iron results in dysbiosis of gut microbiota. Specifically, a promising bacterium ——*Akkermansia*, regarded as the next generation probiotics, was found significantly decreased in HFeD group. *Akkermansia* has been reported to play a probiotic role in easing inflammation and protecting against cancer such as CRC^45^, breast cancer^57^, and others^58^. Considering that iron is required for the growth and virulence of many pathogenic gut bacteria^9^, and dietary iron in the gut could lead to a bloom of pathogenic bacteria in the gut, we suspected that the decrease of *Akkermansia* under dietary iron treatment might be caused by the space occupying inhibition effect from the accumulated pathogenic bacteria.

The mechanisms underlying iron-triggered colorectal carcinogenesis have been demonstrated to include altered WNT signaling as well as gut microbiota dysbiosis. Here, we found that HFeD promoted dysbiosis of the gut bacteria, which led to an increase in SLPI. Mechanically, SLPI released from gut promoted tumorigenesis activated the MAPK signaling pathway, which has been proven to be involved in the pathogenesis^59^, progression^60^ and oncogenesis^61^ of CRC. Bacterial-derived LPS can activate the MAPK signaling pathway ^62,^ which is consistent with the abovementioned dysfunction of intestinal barrier, bacterial translocation, and elevated serum LPS. Supplementation of Akkermansiaceae has been shown to be effective in ameliorating gut barrier dysfunction^63^. In this study, we observed that treating HFeD fed mice with *Akkermansia* repaired the intestinal barrier and reduced the activation of the MAPK pathway.

In conclusion, our study provides the first evidence that excessive dietary iron promotes CRC carcinogenesis by inducing gut microbial dysbiosis. Decreased levels of Akkermansiaceae exacerbate gut barrier dysfunction, leading to bacterial translocation and upregulation of SLPI. Elevated SLPI expression activates the oncogenic MAPK signaling pathway in the epithelium, promoting tumor growth. Importantly, we demonstrate that supplementing Akkermansiaceae can preserve gut barrier integrity and mitigate the tumorigenic effect of high dietary iron consumption.

### Limitations of the study

Although a combination of antibiotics treatment and FMT experiments have been performed to elucidate the effect of dietary iron-modulated gut microbiota, studies using germ-free mice might provide more convincing results. In addition, a prospective clinical cohort study should be carried out in future to verify the higher expression of SLPI and depletion of *Akkermansiaceae* under excessive iron intake.

## Methods

### Animal experiment

Male C57BL/6 mice that were eight weeks old were bought from Gem Pharmatech Guangzhou Co., Ltd. The Sixth Affiliated Hospital of Sun Yat-sen University and Ruige Biotechnology’s animal facilities provided the mice with enough food and drink in specific pathogen-free conditions. All animal experiments were carried out in accordance with the regulations that the Institutional Animal Care and Use Committee (IACUC) of Sun Yat-Sen University and Ruige Biotechnohad approved.

We constructed the AOM/DSS model as described previously.^35^ Briefly, mice were intraperitoneally injected with 10 mg/kg of AOM (MPBIO, 25843-45-2) and administered three cycles of 1% DSS (Meilunbio, MB5535–2) in their drinking water. From the beginning of AOM, mice were fed AIN−93 G (ConD), AIN−93 G with 250 mg/kg of carbonyl iron (LfeD), or AIN−93 G with 1,000 mg/kg carbonyl iron (HFeD) (XIETONG SHENGWU, XT190008). Before harvest, mice were subjected to coloscopic examination using the KARL STORZE endoscopy system. Transgenic APC^min/+^ mice (male, eight-week-old) were purchased from Gem Pharmatech Guangzhou Co., Ltd. Mice were fed ConD, LfeD, or HFeD (XIETONG SHENGWU, XT190008) for 90 days before sacrifice. Antibiotics (ABX)-treated AOM/DSS model mice were handled as above but fed with an additional antibiotics cocktail (ampicillin, Macklin, A830931; vancomycin, Macklin, V820413; metronidazole, Solarbio, M8060; neomycin, Macklin, N814740; ciprofloxacin, Macklin, C861180) every other week to eliminate the effect of microbiota.

To examine the direct effect of iron-modulated microbiota, the local bacteria of C57BL/6 mice were first depleted using the antibiotic cocktail mentioned above for five days. Stool samples stored in 25% glycerol at − 80°C were defrosted and first centrifuged at 70 rcf for 5 min to discard the fecal residue, washed with PBS, and centrifuged three times at 300 rcf to eliminate soluble iron and collect the microbiota. Oral gavage of washed fecal bacteria was performed four times before AOM injection. To examine the effect of *A. muciniphila*, we constructed the AOM/DSS model with oral gavage of 1 × 10^8^ CFU *A. muciniphila* per 3 days and fed with HFeD as previously described.

We injected 1 × 10^6^ tumor cells subcutaneously to one flank of each mouse. For the intertumoral injection experiment, after the tumor diameter of each mouse reached over 5 mm, 100 μl of PBS or SLPI (1 μg/ml) solution was injected into the tumor. The mice were dissected on day 16, and the tumor weight was measured, and specimens were collected. Tumor volume was calculated by the formula V = L * W^2^/2 (L, length; W, width).

Macroscopic neoplastic lesions were carefully identified and counted upon sacrifice. The tumor size was measured using an electronic digital caliper. Sections measuring approximately 0.5 × 0.5 cm were collected from each mouse, snap-frozen in liquid nitrogen, and stored at − 80°C. The remaining intestinal tissue was Swiss-rolled and formalin-fixed for histological examination.

### Cell culture

The CT26 and HIEC6 cell lines used in this study were purchased from the American Type Culture Collection. The cell lines were cultured in Gibco DMEM (GIBCO, 11966025) with 10% fetal bovine serum (ExCell Bio, FSP500) and 1% penicillin – streptomycin (GIBCO, 15140–122) at 37°C in 5% CO_2_ and 95% humidity.

The fecal microbiota were isolated by centrifugation, as described above. Recombinant mouse SLPI (cloud-clone, RPB312Mu01–200) was added (1 μg/ml) to the culture medium for three days.

### Bacteria culture

The isolation of *A. muciniphila* were performed by anaerobically culturing the fecal microbiota in BHI plate for 5 days. Then, clones were patched and performing 16S rRNA gene sequencing. *A. muciniphila* was cultured in BHI plate with 4 g/L mucin (Sigma,84082-64-4) for 5 days in anaerobic atmosphere for consecutive experiments.

### Western blot

Utilizing Biosharp’s RIPA-BL504A, total protein from the colonic epithelium was extracted, followed by SDS-PAGE and transmembrane to PVDF. The membranes were incubated with claudin−3 (Abcam, ab214487), claudin−8 (Bioss, bs−5016 R), slpi (Novus, NBP1–76803), and GAPDH (Abcam, ab181602) after being blocked with 5% milk (Biofroxx, 68514-61-4). The membranes were subsequently incubated with secondary antibodies and Western blotting detection agents that enhanced chemiluminescence (Solarbio, PE0010). The levels of claudin−3, claudin−8, and SLPI were normalized to GAPDH intensity. ImageJ was used to calculate protein band intensities.

### Quantitative reverse-transcription PCR (qRT-PCR)

Colon tissue was homogenized by tissue homogenizer (LUKYM-I) and total RNA was isolated using the FastPure Cell/Tissue Total RNA Isolation Kit (Vazyme, RC112–01), and the HiScript III 1st Strand cDNA Synthesis Kit with a gDNA wiper (Vazyme, R312–01) was used to reverse-transcribe the RNA into cDNA. The QuantStudio^TM^ 7 Flex Real-Time PCR System (Thermo Fisher Scientific) was used to measure the relative expression level of the gene and normalize it to the expression level of β-actin. Primers used in this study are listed in Supplementary Table 1.

### Serum LPS quantification

Serum LPS levels were measured using an ELISA kit (KT37561; MSKbio). All testing procedures were performed according to the manufacturer’s instructions.

### 16S ribosomal RNA gene sequencing and sequence curation and annotation

The V4 variable region was amplified by PCR using dual barcoded primers for 16S rRNA gene sequencing (338F: 5’-ACTCCTACGGGAGGCAGCA−3’; 806 R: 5’-GGACTACHVGGGTWTCTAAT−3’). The Quant-iT PicoGreen ds DNA Assay Kit (Thermo Fisher Scientific) was used to quantify the amplicons after they were purified with AMPure XP (Beckman Coulter). A Bioanalyzer 2100 with the High Sensitivity DNA Kit (Agilent) and the KAPA Library Quantification Kit for Illumina (Kapa Biosystems) were used to further qualify and quantify the pooled amplicons. The denatured amplicons were blended in with 20% PhiX Control v.3 and sequenced on a HiSeq (Illumina, 2 × 250-bp matched end peruses). The samples were quantified using an open reference-based 16S rRNA gene amplicon sequence variants (ASVs) pickup strategy. Demultiplexing and quality filtering were carried out with the help of the QIIME2 platform’s recommended parameters. After that, the ASVs formed from the 100% similar 16S V4 sequences. Based on the GreenGene database, the UCLUST algorithm was used to assign an ASV’s taxon. To avoid sampling depth bias, a random selection of 10,000 tags was chosen for each sample. The relative abundance of each ASV was calculated as a proportion of the total sequences in each sample.^64^

### Immunofluorescence assay

Paraffin-embedded colon sections were de-paraffinized, antigen retrieved, blocked, and incubated with the antibody SLPI (Novus, NBP1–76803). The slides were then incubated with DAPI and examined under a laser scanning confocal microscope (Zeiss, Germany).

### Fluorescence in situ hybridization (FISH)

A CY3-conjugated EUB338 universal bacterial probe (GCTGCCTCCCGTAGGAGT) was labeled with Spectrum-Red (Focobio). Then, 5-μm-thick paraffin sections were deparaffinized, incubated in lysozyme solution, washed, hybridized (35% hybridization buffer) with the FISH probe, incubated for 24 h, washed, stained with DAPI, and imaged using a fluorescent microscope system (Zeiss, Germany）.

### Slpi gene silencing

In accordance with the manufacturer’s instructions, the shslpi no. 1 (GAAAGTCTGCCACCTACTTCTCGAGAAGTAGGTGGCAGACTTT), the shslpi no. 2 (GATGCTTAACCCTCCCAATGTCTCGAGACATTGGGAGGGTTAAGCAT), and the shNT (shGFP TRCN0000072181) were co-transfected with plasmids encoding psPAX2 and pMD2.G using PEI MAX 40 K to generate lentiviruses. After transfection, the medium was changed every 24 hours, and the virus was harvested, filtered, and used to infect CT26 cells for 48 hours with 1 g/ml of polybrene. Puromycin was used to select resistant colonies for 36 hours.

### RNA sequencing and data analysis

Trizol® Reagent (Life Technologies, Carlsbad, CA, USA) was used to extract total RNA from intestinal tissues, and isopropanol was used to precipitate the RNA. BioAnalyzer analysis and agarose gel electrophoresis were used to assess the integrity of the RNA. The TruSeq RNA Sample Prep kit (Illumina, San Diego, CA) was used to prepare the paired-end, non-strand-specific RNA-seq libraries, which were successfully constructed from samples. At least 100 ng of the total RNA was transcribed into cDNA for the RNA-seq libraries, and the Nanodrop instrument (Thermo-Fisher) was used to measure the number of cDNA libraries. After that, we produced sheared cDNA with a covaries S220 system (Covaris, Woburn, MA), quantified the cDNAs once more with Nanodrop, and determined the appropriate quantity of cDNA for the construction of the library. Each cDNA library contained sample-specific barcodes, which were then pooled and sequenced using a 2100bp HiSeq run on a HiSeq platform (illumina, San Diego, CA). The DESeq2 R package (1.16.1) was utilized for differential expression analysis of two groups (two biological replicates per condition). Using a model based on the negative binomial distribution, DESeq2 provides statistical procedures for determining differential expression in digital gene expression data. The Benjamini and Hochberg method was used to adjust the resulting p-values to control the rate of false discovery. Differentially expressed genes 66 were identified by DESeq2 with an adjusted p-value of less than .05.

### Quantification and statistical analysis

Barring the factual investigation referenced over, any remaining measurable examinations were performed utilizing GraphPad Prism 8. Information is introduced as the mean ± SD, and contrasts between two gatherings were looked at utilizing matched or unpaired Student’s t-tests and Mann-Whitney U-tests. The Kruskal – Wallis test or one-way ANOVA were used to compare three or more groups. Differences with p-values less than 0.05 were deemed significant (*p < .05, **p < .01, and ***p < .001, ****p < .001), whereas ns indicated comparisons that were not significant. All p-values were two-tailed.

## Supplementary Material

Supplemental Material

## Data Availability

The authors confirm that the data supporting the findings of this study are available within the article and its supplementary materials. The 16S rDNA-sequencing data was deposited in the Sequence Read Archive (SRA; https://www.ncbi.nlm.nih.gov/sra) under the accession number: PRJNA907929.
