# Supplementary material for: Dietary iron modulates gut microbiota and induces SLPI secretion to promote colorectal tumorigenesis
Source: Gut Microbes. 2023 Jun 13;15(1):2221978. doi: 10.1080/19490976.2023.2221978 (PMC10269393; doi:10.1080/19490976.2023.2221978)
Supplement: Supplemental Material [file KGMI_A_2221978_SM1297.zip › Supplemental Material/Supplementary figure legends clean.docx]

Supplementary data

**Figure S1 Proper dietary iron did not increase colorectal tumorigenicity in mice.** (a) Schematic overview of the AOM/DSS-induced cancer model. Mice were fed ConD or LFeD for 10 weeks. AOM (10 mg/kg) was injected intraperitoneally at day 4. Mice were sacrificed at the end of week 10 (ConD group, n=6; LFeD group, n=6). (b) Tumor number in the mice of ConD and LFeD groups. (c–d) Representative images of colonoscope, colon, H&E staining, and Ki67-positive cells of colon sections in the ConD group and LFeD group. Scale bars, 250 μm. Data are expressed as the mean±SD. Statistical significance was determined by unpaired Student’s t-test.

**Figure S2 ABX treatment eliminates the effect of excessive dietary iron on tumorigenesis.** (a) Schematic overview of the AOM/DSS-induced cancer model. Mice were fed a control diet (ConD+ABX) or high-iron diet (HFeD+ABX) for 10 weeks and given antibiotics cocktail periodically. AOM (10 mg/kg) was injected intraperitoneally at day 4. Mice were sacrificed at the end of week 10 (ConD+ABX group, n=6; HFeD+ABX group, n=6). (b) Tumor number in the mice of ConD+ABX and LFeD+ABX groups. (c) Representative images of H&E staining and Ki67-positive cells of colon sections in the ConD+ABX group and HFeD+ABX group. Scale bars, 250 μm. (d) Quantitation of Ki67 expression in colons. Data are expressed as the mean ± SD. Statistical significance was determined by unpaired Student’s t-test.

**Figure S3** **The mouse colon transcriptome indicates gut barrier destruction.** (a) KEGG pathway enrichment assay of the mouse transcriptome. (b) Gene expression of CDH1, CLDN1, CLDN2, CLDN3, CLDN8, and ZO-1 in mice from ConD-FMT (n=11) and HFeD-FMT (n=12) groups. Data are expressed as the mean ± SD. Statistical significance was determined by multiple unpaired Student’s t-test. * *p* <0.05, ** *p* <0.01, *** *p* <0.001, **** *p* <0.0001.

**Figure S4 SLPI is important in tumor development.** (a) Gene expression of SLPI in mice colon from ConD (n=5) and HFeD (n=5) mice (AOM/DSS model) and ConD-FMT (n=11) and HFeD-FMT (n=12) mice (AOM/DSS model). Presented with 3 independent experiments. Statistical significance was determined by nested ANOVA. (b) Protein expression of SLPI in mice colon from ConD (n=5) and HFeD (n=5) mice (AOM/DSS model). Presented with 3 independent experiments. Statistical significance was determined by nested ANOVA. (c) Protein expression of SLPI in the colon of mice from ConD and HFeD groups (Apc^min/+^ model) determined by western blot. For protein expression analysis, we randomly selected six mice from each group for analysis. (d) Protein expression of SLPI in mice colon from ConD-FMT and HFeD-FMT mice (AOM/DSS model). Presented with 3 independent experiments. For protein expression analysis, we randomly selected five mice from each group for analysis. Statistical significance was determined by nested ANOVA. (e-f) Images and quantitation of SLPI expression in the colon of mice from ConD+ABX and HFeD+ABX groups (AOM/DSS model) by western blot (left) and qRT-PCR (right). (g) Quantifications of IF of SLPI in mice colon from ConD and HFeD mice (AOM/DSS model) and ConD-FMT and HFeD-FMT mice (AOM/DSS model). For IF analysis, we randomly selected five mice from each group for analysis. Presented with 3 independent experiments. Statistical significance was determined by nested ANOVA. (h) Schematic overview of the in vivo tumor growth model for mice subcutaneously injected with CT26 and intratumorally injected with PBS (NC) or SLPI. (i) Schematic overview of the in vivo tumor growth model for mice subcutaneously injected with CT26 pre-treated with PBS (NC) or SLPI for two days. (j) CCK8 assay of CT26-shNC, CT26-shSLPI#1, Ct26-shSLPI#2. (k–m) In vivo primary tumor growth (k), representative images and statistical chart (l; m) in mice subcutaneously injected with CT26-shNC, CT26-shSLPI#1, and Ct26-shSLPI#2. (n) PCA comparing CT26 transcriptomes between PBS-treated and SLPI-treated mice. Statistical significance was determined by unpaired Student’s t-test and ordinary one-way ANOVA with Tukey’s multiple comparisons. * *p* <0.05, ** *p* <0.01, *** *p* <0.001. IF, immunofluorescence.

**Figure S5** **SLPI significantly activates the SAPK/JNK signaling pathway.** (a) gene set enrichment analysis (GSEA) comparing CT26 treated with PBS or SLPI (n = 3 per group). KEGG pathways are shown. (b) Protein expression of p-SAPK/JNK and SAPK/JNK in CT26 treated with PBS or SLPI (n = 3 per group). (c) Protein expression of p-Erk1/2, Erk1/2, p-p38, and p38 in CT26 treated with PBS or SLPI (n = 3 per group). (d–e) Protein expression of phosp-SAPK/JNK in mice from ConD and HFeD groups (d) or mice from ConD-FMT and HFeD-FMT groups (e) determined by western blot.

**Figure S6 Dietary iron alters gut microbiota composition.** (a) Alpha diversity (ACE, Shannon) boxplot of mice in ConD (n=6) and HFeD (n=6) groups. (b) Alpha diversity (ACE, Shannon) boxplot of mice in ConD-FMT (n=11) and HFeD-FMT (n=12) groups. (c) Alpha diversity (simpson index) boxplot of mice in ConD (n=6) and LFeD (n=6) groups. (d) PCA comparing mouse microbial compositions in fecal samples between ConD and LFeD groups. (e) LDA score computed from features with differential abundance between ConD and LFeD groups. Data are expressed as the mean ± SD. Statistical significance was determined by unpaired Student’s t-test. * *p* <0.05, ** *p* <0.01.

**Figure S7 Rarefaction curve of 16S rRNA sequencing of mice fed with HFeD (a) and FMT (b).**

**Figure S8 Sketch map of the mechanism by which excessive dietary iron modulates gut microbiota and promotes tumor development.** Created by Biorender.com
