# Supplementary figures and images for: Dietary iron modulates gut microbiota and induces SLPI secretion to promote colorectal tumorigenesis
Source: Gut Microbes. 2023 Jun 13;15(1):2221978. doi: 10.1080/19490976.2023.2221978 (PMC10269393; doi:10.1080/19490976.2023.2221978)

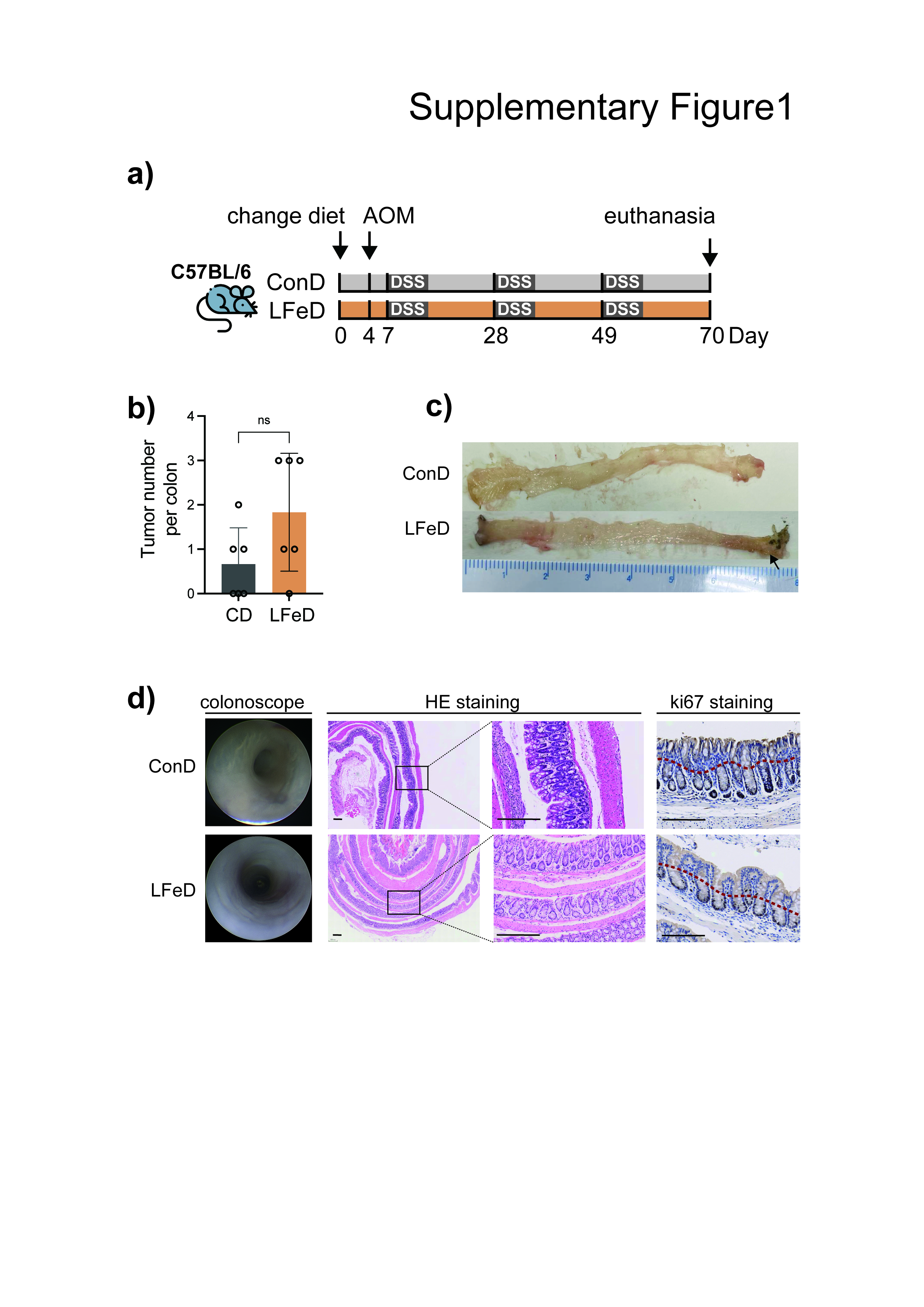

Supplement: Supplemental Material [file KGMI_A_2221978_SM1297.zip › Supplemental Material/Figure_S1.tif]

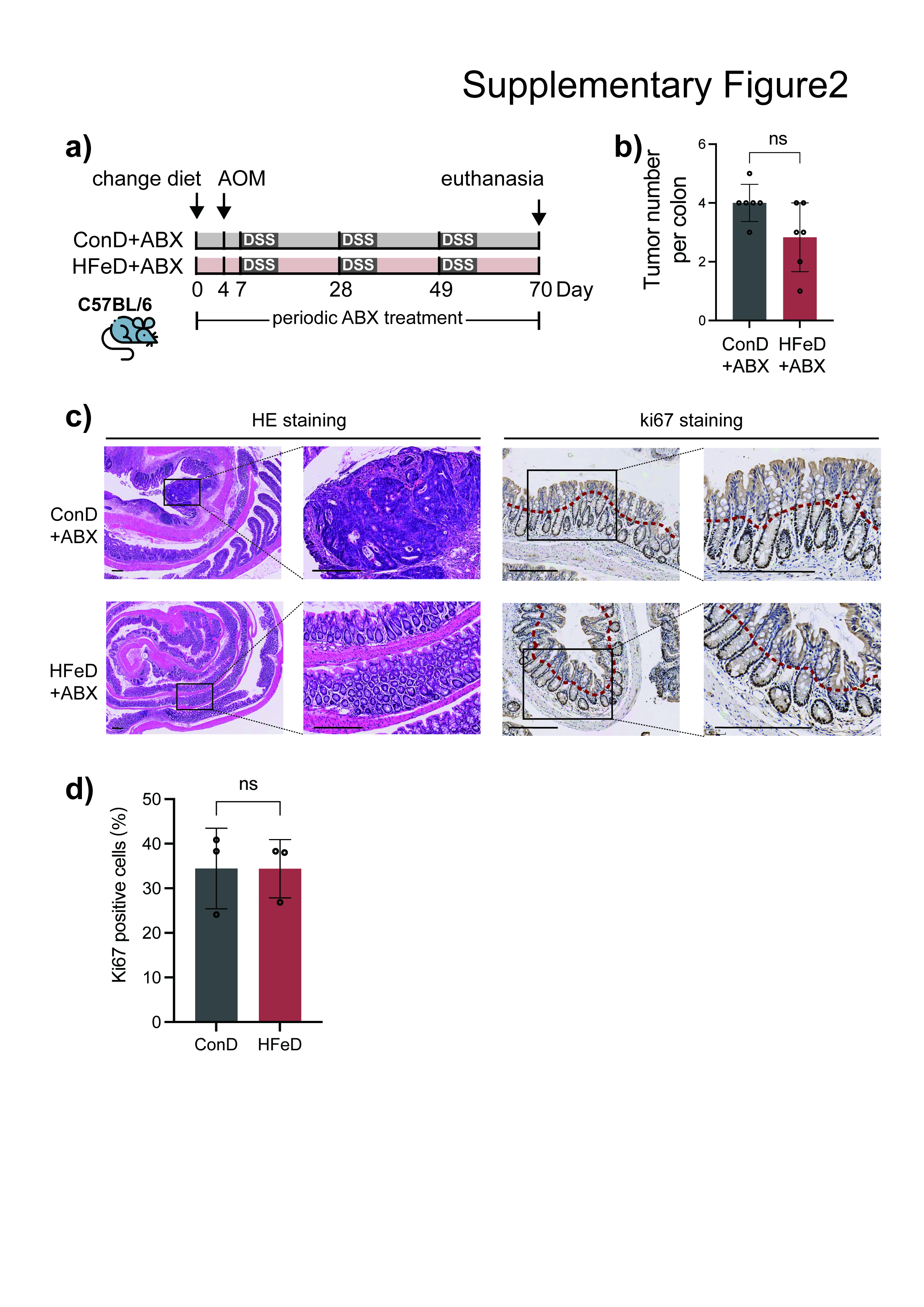

Supplement: Supplemental Material [file KGMI_A_2221978_SM1297.zip › Supplemental Material/Figure_S2.tif]

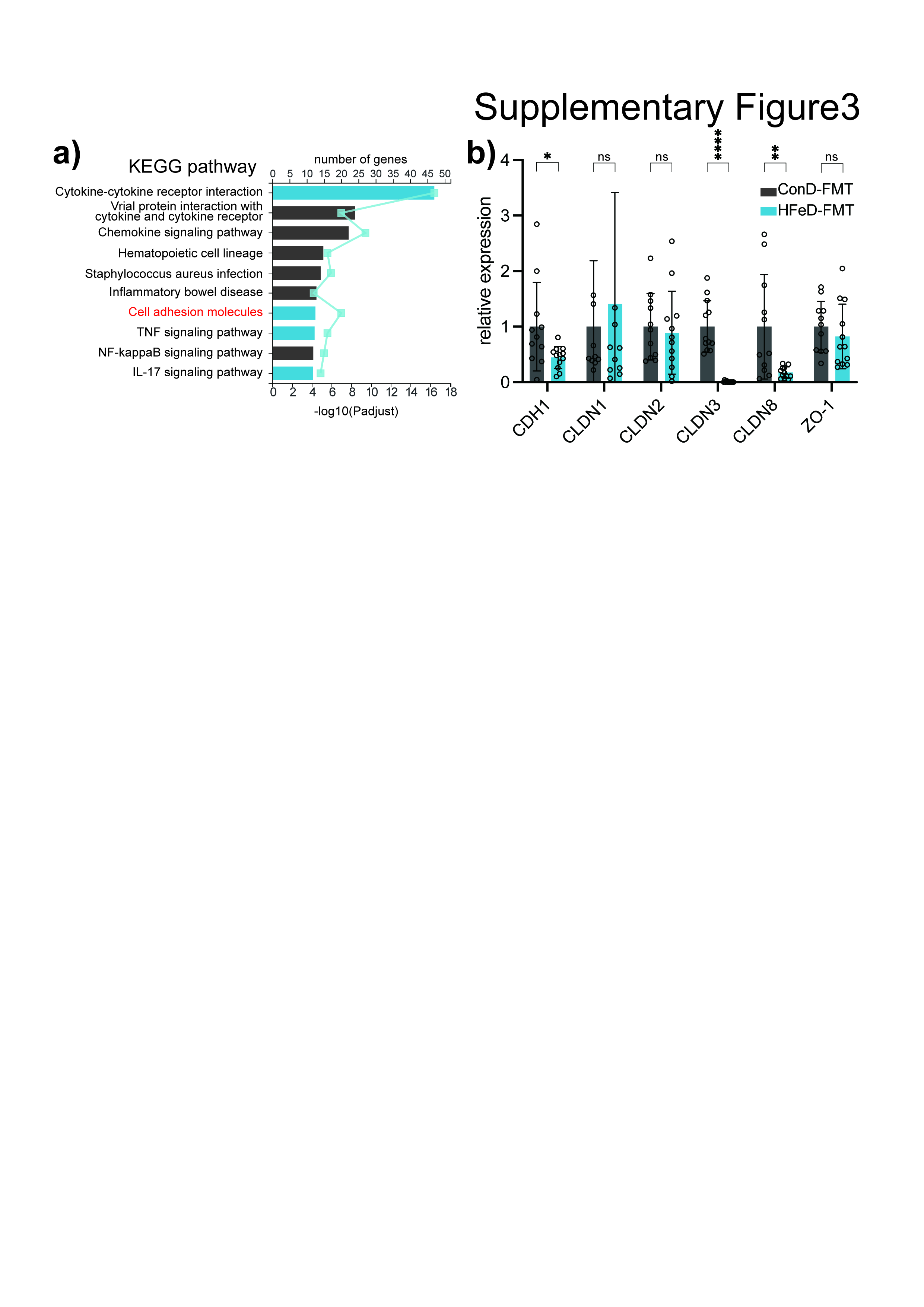

Supplement: Supplemental Material [file KGMI_A_2221978_SM1297.zip › Supplemental Material/Figure_S3.tif]

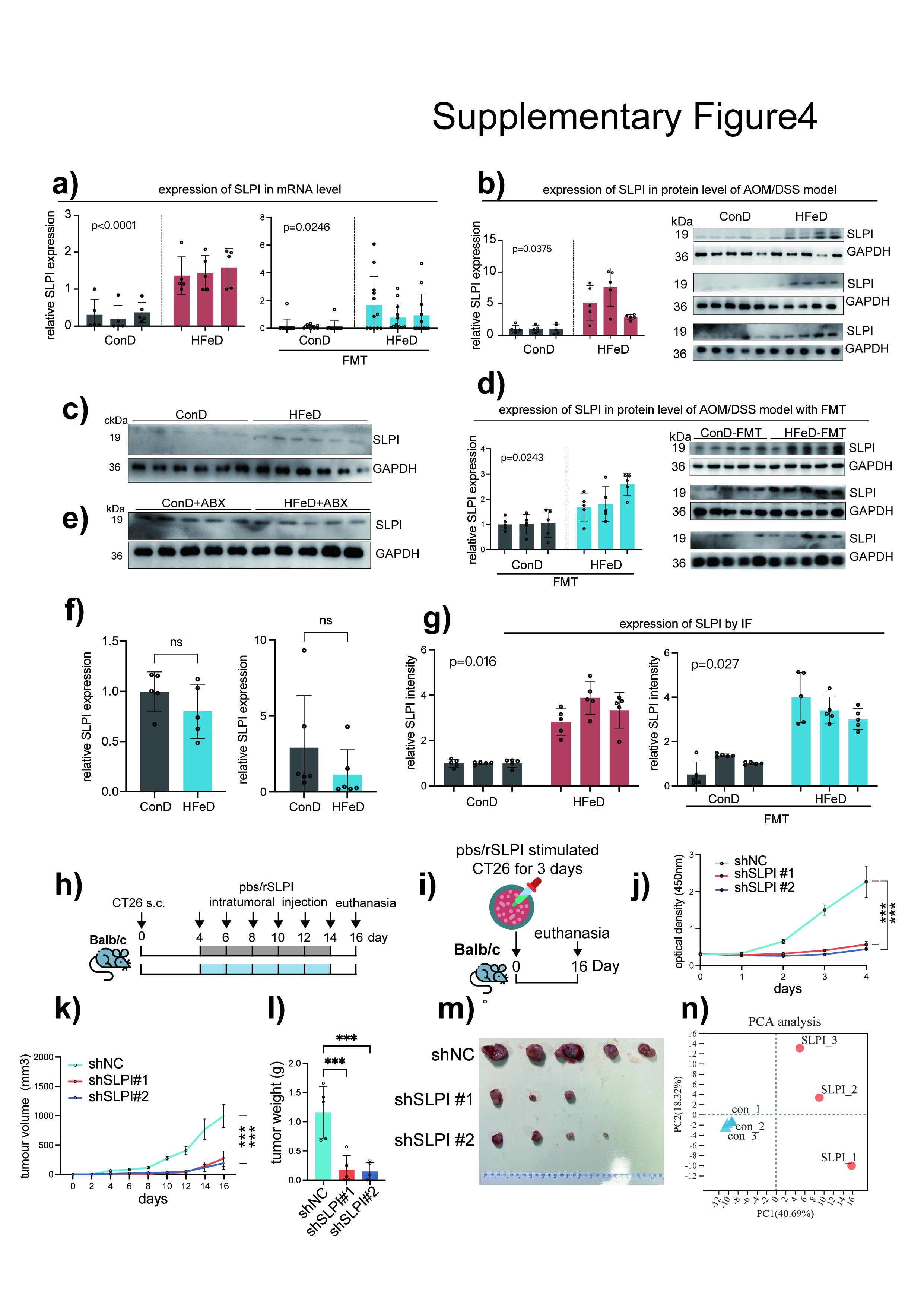

Supplement: Supplemental Material [file KGMI_A_2221978_SM1297.zip › Supplemental Material/Figure_S4.tif]

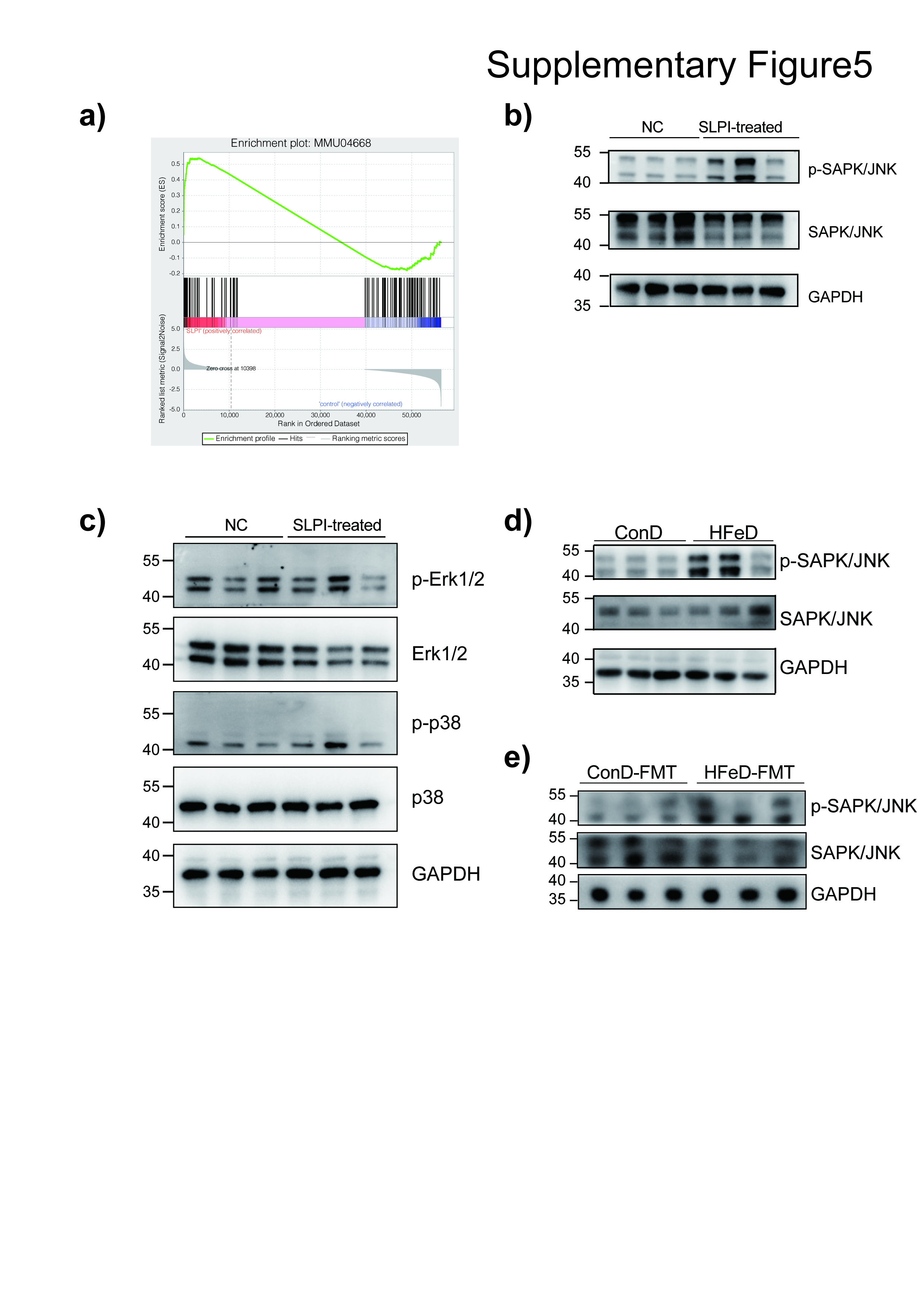

Supplement: Supplemental Material [file KGMI_A_2221978_SM1297.zip › Supplemental Material/Figure_S5.tif]

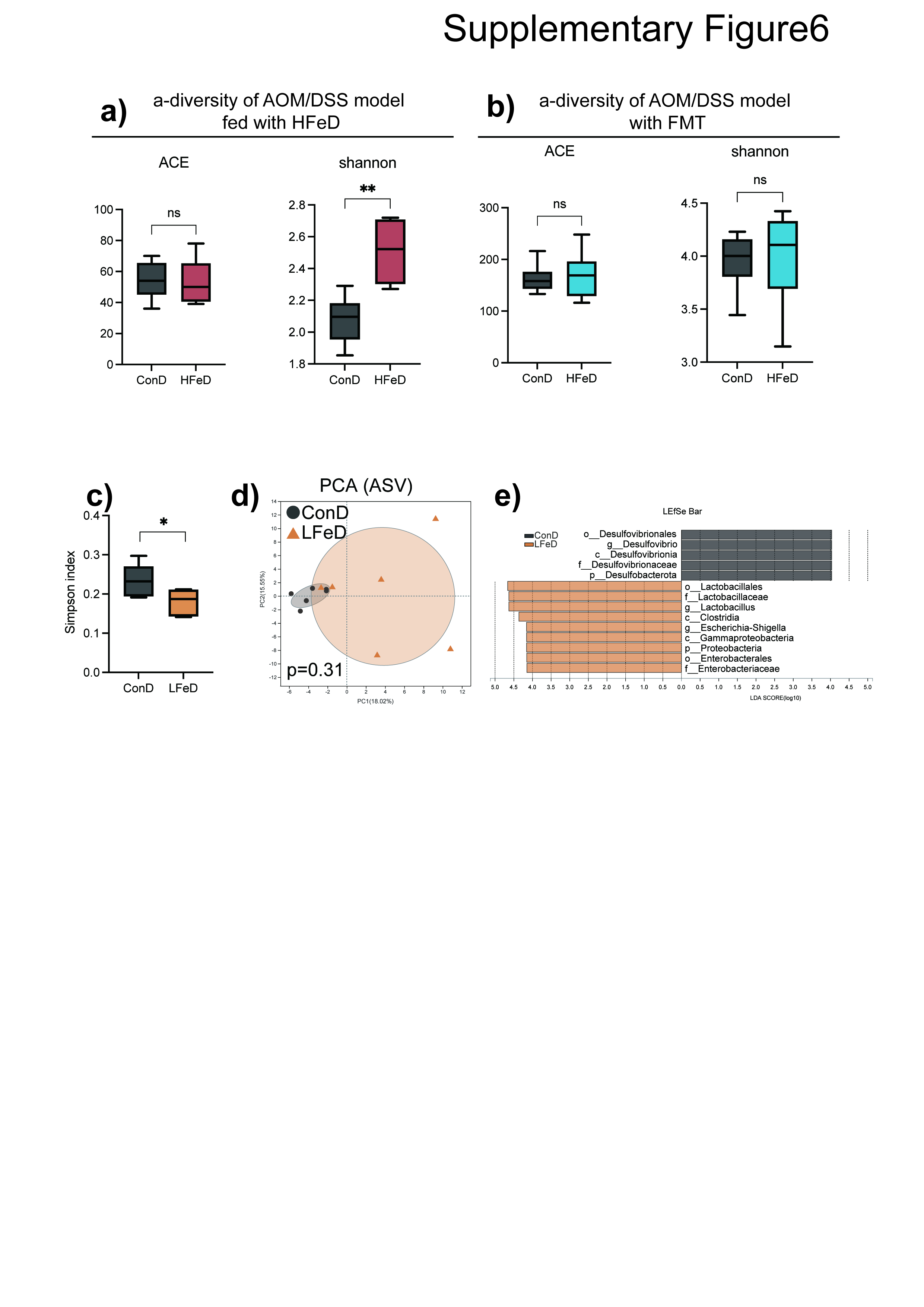

Supplement: Supplemental Material [file KGMI_A_2221978_SM1297.zip › Supplemental Material/Figure_S6.tif]

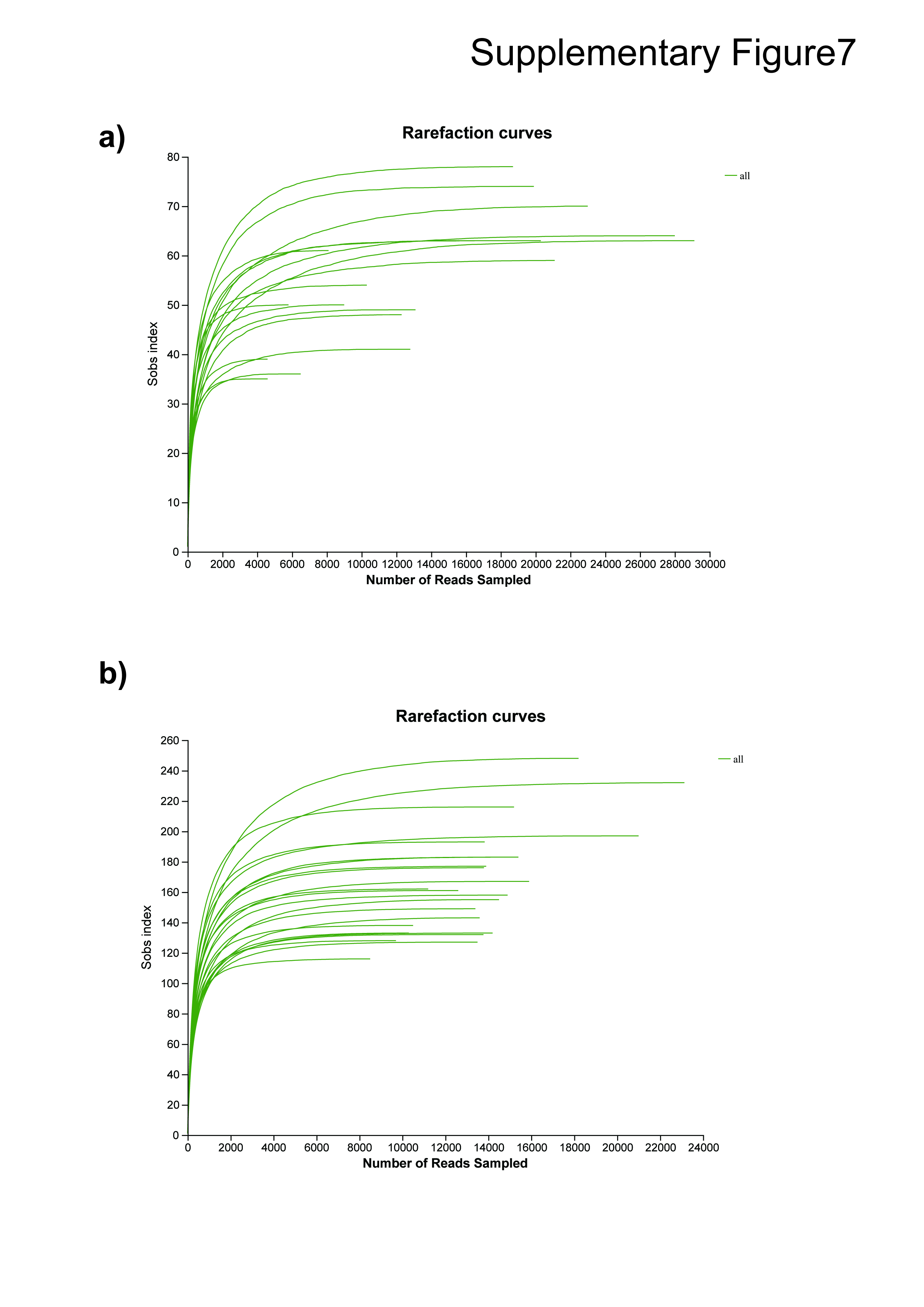

Supplement: Supplemental Material [file KGMI_A_2221978_SM1297.zip › Supplemental Material/Figure_S7.tif]

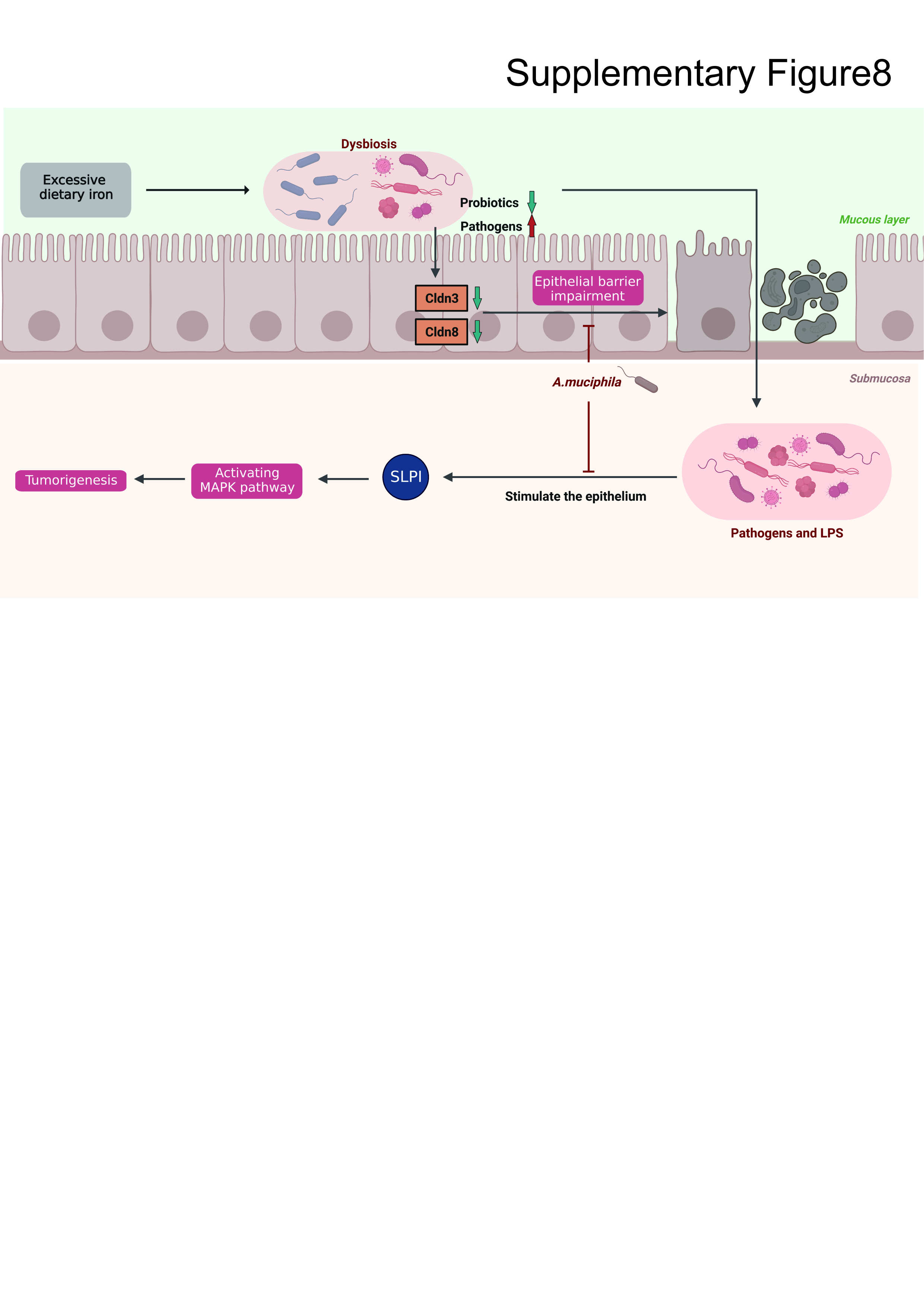

Supplement: Supplemental Material [file KGMI_A_2221978_SM1297.zip › Supplemental Material/Figure_S8.tif]
